# Clustering of children’s oral diseases in families and villages in a rural setting in Egypt

**DOI:** 10.1186/s12903-023-02922-2

**Published:** 2023-04-27

**Authors:** Maha El Tantawi, Amira H. Elwan, Hams Hamed

**Affiliations:** Department of Pediatric Dentistry and Dental Public Health, Faculty of Dentistry, Alexandria University, Champollion St, Azarita, 21527 Alexandria Egypt

**Keywords:** Dental caries, Gingivitis, Dental plaque index, Multilevel analysis, Child, Toothbrushing

## Abstract

**Background:**

This study assessed the clustering of children’ caries experience, plaque accumulation and gingival inflammation in families and villages in Northwestern Egypt and the factors related to the severity of these conditions.

**Methods:**

This was a secondary analysis of a 2019 household survey of children in villages around Alexandria, Egypt. Clinical examination assessed primary and permanent teeth caries experience (dmft/ DMFT using the World Health Organization criteria), plaque accumulation (Plaque Index (PlI)) and gingival inflammation (Gingival Index (GI)). A child questionnaire assessed child’s age, sex, the frequency of toothbrushing (at least twice daily versus less) and frequency of consuming eight types of sugary products (daily sugar consumption score, sum of sugary products consumed daily). Mothers’ questionnaire assessed the number of children in the family, mother’s education (at least high school versus less), at least twice daily toothbrushing and daily sugar consumption similar to the child. Multilevel regression analyses assessed clustering, calculated by the intraclass correlation coefficient (ICC) of the three conditions in families and villages. Regression estimates (B) and 95% confidence intervals (CIs) of individual and family factors were calculated.

**Results:**

Complete data were available for 450 children (246 families, seven villages], mean = 9.9 years-old and 56% females. The mean caries experience score = 3.6, mean plaque index = 1.5 and mean gingival index = 1.2. Caries experience, plaque accumulation and gingival inflammation were not clustered in villages (ICC < 0.01) but clustered in families (ICC = 0.10, 0.44 and 0.29). Child factors significantly improved model fit for caries experience and gingivitis (p < 0.001) but not plaque accumulation (p = 0.90). Family factors did not improve any model fit (p > 0.05). Child’s age was significantly associated with caries experience (B= -0.48, p < 0.001) and gingival inflammation (B = 0.032, p < 0.001). Children who brushed their teeth twice daily had significantly more caries experience (B = 1.04, p = 0.01).

**Conclusion:**

The three oral conditions were not clustered in villages but clustered in families. Plaque accumulation showed the greatest within-family clustering. Family factors were not associated with the three conditions and individual factors indicated the need for interventions to promote preventive behaviors and identify families at risk of oral conditions.

## Introduction

Oral health is an integral component of general health and a major determinant of the quality of life. Oral diseases are among the most prevalent globally affecting a large proportion of the global populations [1]. In the Middle East and North Africa region, dental caries in children is associated with older age, female sex, frequent sugar consumption, inadequate toothbrushing, accumulation of dental plaque, low socioeconomic status, and irregular dental visits [2]. Gingivitis is another oral disease that affects children. It is associated with poor oral hygiene, plaque accumulation, lower socioeconomic status, less oral health knowledge and skills of tooth cleaning [3].

Individual factors do not fully explain disparities in oral health and contextual determinants should be taken into consideration. Fisher-Owens et al. proposed a conceptual model to explain how determinants at different levels may affect children’s oral health including genetic and biological factors, social environment, physical environment, health behaviors, and access to health care. These factors interrelate at community, family, and individual levels to affect children’s oral health and account for differences in oral health among children living in the same and different environments [4].

Research shows the impact of family and parents especially mothers on oral health. The quantity–quality trade-off theory indicates that an increase in the number of children in a family may decrease available resources for investing in human capital per child leading to a trade-off between quantity and quality of children so that the number of siblings may be negatively associated with child health and health expenditure [5]. Having two or more siblings was associated with lower odds of regular brushing and annual dental visits [6]. Mothers with high educational levels were found to have children who were less affected by oral diseases whereas mothers with low literacy had less oral health knowledge and less healthy practices [7]. Mother’s toothbrushing [8] and frequent consumption of unhealthy food [9] were associated with children’s caries experience.

Studies on the association between children’s oral health and the environment beyond the family that were conducted in higher income countries such as Brazil, North America and Japan may not apply to countries with lower income. These studies also mostly focus on mostly urban and large administrative areas such as census tracts [10, 11] or administrative divisions of cities [12, 13] which differ from less industrialized and more cohesive small rural communities. Most existing studies do not provide direct comparison between the impact of factors at the level of the individual, family and community. Assessing this relative impact is needed to guide policy setting and the design of oral health promotion interventions. For example, in closely knit rural settings, community attributes may have a powerful effect on health behaviors, and interventions targeting these collective attributes may succeed in promoting healthy behaviors than elsewhere. Family characteristics may also affect individual inclinations to a great extent in rural communities [14]. A greater focus is needed on the interaction of individual, family and community factors on children’s oral health in rural, less urbanized and lower income settings. The global oral health status report [1] shows that the greatest percent increase in cases of caries in deciduous and permanent teeth as well as cases of periodontal diseases is in low and lower income countries as well as Africa and the Eastern Mediterranean region. The report also shows that the estimated prevalence of various oral diseases in Egypt is among the highest in countries in the Middle East and North African. However, there is inadequate information about the impact of family characteristics versus individual’s choices and differences by community on children’s oral health in rural communities.

The aim of the study was to assess the within family and village clustering of caries experience, plaque accumulation and gingival inflammation in children in a rural setting in Egypt and to identify the factors associated with the severity of these oral diseases and conditions. The hypothesis was that there would be greater differences between than within villages and families in the three oral conditions due to the greater similarity and clustering of these oral conditions within these entities since they share contextual factors.

## Methods

In this study, we conducted a secondary analysis of data primarily collected in a cross-sectional household survey to assess the association between parenting practices and oral health in children in rural areas in Alexandria, Egypt in 2019 [14]. Ethical approval was obtained from the Research Ethics Committee at the Faculty of Dentistry Alexandria University (IRB 00010556 – IORG 0008839). Participants were informed they were free to participate and could withdraw at any time without any negative consequences. The study was conducted in full accordance with the Helsinki Declaration. Informed consent was given before participation.

Children were included if they were older than 6 years of age, free from medical and intellectual disabilities, living in the villages included in the study, if parents consented to their participation, and if they lived with their mother/ female caretaker at the same household. Sample size for the original study was calculated based on 95% confidence level to detect caries among Egyptian children of a mean (SD) DMFT = 1.04 (1.56), mean (SD) deft = 4.21 (3.21) [14, 15]. The required number of children was calculated to range from 366 to 370 children.]. Children were included using multi-stage random sample technique to select a rural administrative center in Northwestern Delta around Alexandria then villages then households in villages. In the first stage, a rural administrative center was selected followed in the second stage by random selection of four villages from the 28 rural units in this center. The number of villages ensured that the required sample size would be attained. In the third stage, a village local guide helped randomly select eligible homes in each village. In the last stage, all eligible children per household were selected [16].

Data were collected using a questionnaire and clinical examination. The questionnaire was based on the Arabic translation of the World Health Organization (WHO) child questionnaire [17]. The first section of the questionnaire collected child’s information and was directed to the child. It assessed the child’s sex, age in years on the last birthday, frequency of toothbrushing (categorized into never, 2–3 times a month, once a week, 2–6 times a week, once a day and 2 or more times a day) and the frequency of consuming eight types of sugary foods/ products (categorized into several times a day, once every day, several times a week, once a week, several times a month and never). The 2nd section was based on the WHO adult questionnaire [18] after its translation to Arabic for use in the present study followed by back translation to English to ensure accuracy. It was directed to the mother asking about her information. It assessed the number of siblings in the household, the mother’s education (categorized into no formal education, less than primary school, primary school completed, middle school completed, high school completed and college or university completed). There were also questions assessing the frequency of toothbrushing and the frequency of consuming the same types of sugary foods or products as for the child. To minimize variation and ensure the accuracy of data collection, interviewers [AHE, HH] were trained on how to present the questionnaire to participants, obtain and record responses.

The clinical examination assessed plaque accumulation and gingival inflammation using the Plaque index of Loe and Silness [19] and the gingival index of Silness and Loe [20] on six index teeth on a 4 points Likert scale ranging from none (0) to 3, severe gingivitis marked by spontaneous bleeding (for the Gingival index) and heavy plaque accumulation manifested as abundance of plaque on the gingival margin (in case of the Plaque index). Person level score was the average of the six scores of all index teeth. Caries experience was also assessed in the primary (dmft) and permanent (DMFT) teeth. Each tooth was classified as sound; decayed if there was cavitation into dentin on any surface, missing due to caries or filled if it had any permanent restoration following the WHO criteria [18]. The examination was conducted using a blunt probe and mouth mirror under natural daylight with participants seated on a chair. No radiographic examination, magnification, or drying were used. The examination was conducted by 2 examiners [NMA and RY] who received training for caries diagnosis by a gold standard examiner followed by assessment of inter-examiner agreement and analysis that showed excellent inter-examiner agreement (Kappa = 0.91).

After explaining the study purpose and obtaining consent, the mother was interviewed by a researcher. At the same time, another researcher examined the eligible children and their clinical oral health status was recorded. After the examination, the children were interviewed separately and individually away from the mother so that their responses would not be affected by her reactions. Data were entered into the online open-source platform, Kobotoolbox which allowed data entry offline in the field and synchronization later when internet access became available.

Data were downloaded, cleaned and exported to SPSS V.25 for analysis. The frequency of brushing was recoded into brushing twice or more daily with yes (brushing twice or more daily) and no (all the other categories). A score of daily sugar consumption was created by counting the number of the eight sugary foods/ products that participants reported to consume at least once daily, and the score ranged from zero (none of the products consumed daily) to 8 (all products consumed daily). Mother’s education was categorized into has at least secondary education (including secondary education and university education) and less than secondary education (including no formal education, less than primary school completed, primary school education and middle school education). We used the WHO cutoff points to classify children who were 12 years and older into groups based on caries experience severity as follows: very low: 0.1–1.1, low: 1.2–2.6, moderate: 2.7–4.4, high: 4.5–6.5 and very high:> 6.5 (18). Descriptive statistics were calculated as means and standard deviation for quantitative variables and frequencies and percentages for categorical variables- for the whole sample and for the subgroup of participants who had siblings. Further analysis was restricted to those with siblings.

**Fig. 1:**
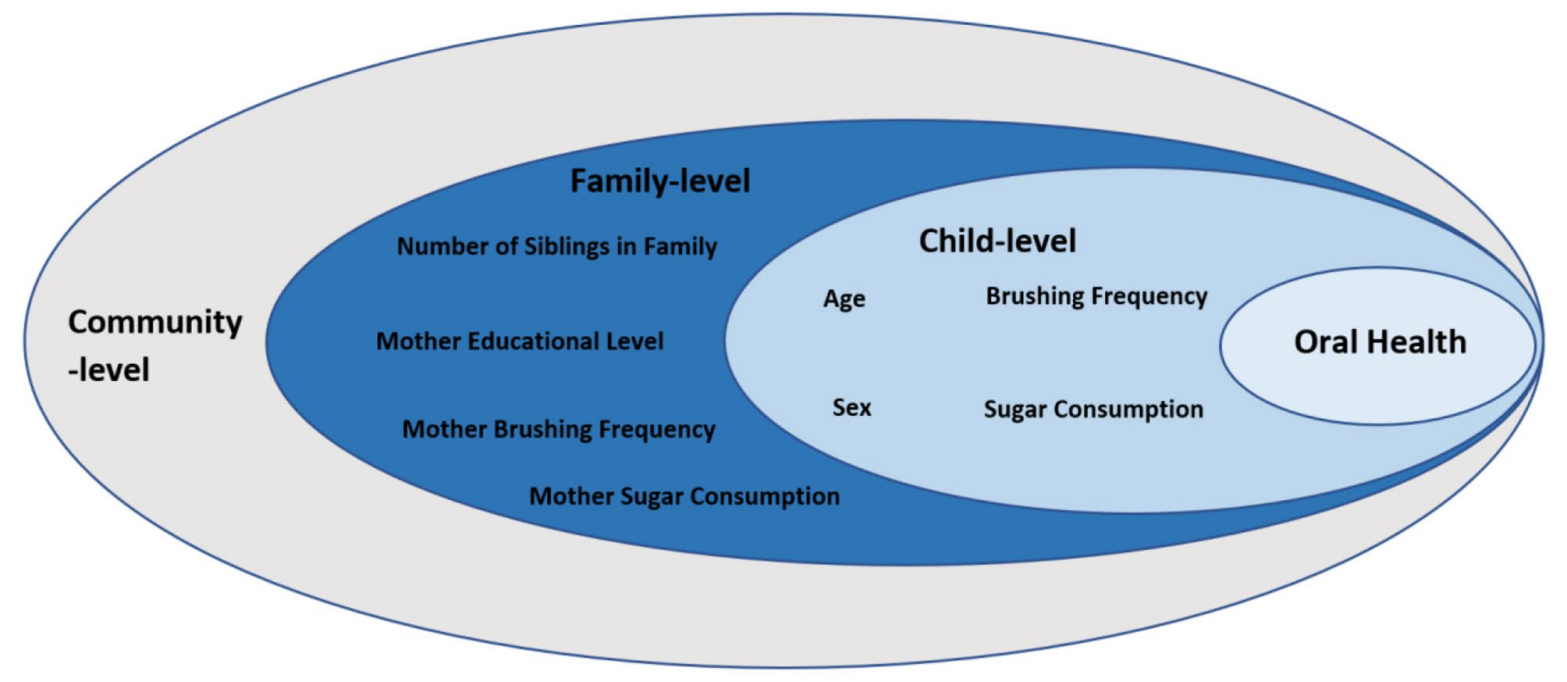
Child and family level factors in the study aligned with the Fisher Owens model. Community level factors are in grey, indicating they are not included in the study

There were three dependent variables: the number of teeth with caries experience (dmft/ DMFT), the plaque index score (PlI) and the gingival index score (GI). Because children were clustered in families and families were clustered in villages, we used multilevel linear multiple regression analysis in 5 steps for each dependent variable. First, an unconditional model was developed where the random effects of both villages (level 3) and families (level 2) were considered to allow a direct comparison of the magnitude of clustering in both levels. In the second step, only families as level 2 random effects were included due to the minimal clustering at village level (reported in Results below). In these two steps, no fixed effect variables were included. The independent variables included in subsequent steps are seen in Fig. 1. In steps 3–5 we developed conditional models by including fixed effect factors with variance components covariance type. In step 3, we added fixed effect variables for level 1 (child): child sex, age, whether the child brushed their teeth twice daily, and daily sugar consumption score. In step 4, we removed the level 1 fixed effect variables and replaced them with fixed effect variables at level 2 (family): number of siblings, whether the mother had at least secondary school education, whether she brushed her teeth twice daily, and the daily sugar consumption score of the mother. In step 5, we included level 1 and 2 factors as fixed effects. For each model, we calculated the model deviance calculated as − 2 log likelihood (-2LL). We also calculated level 1 and 2 variances and used these to calculate the intra-class correlation coefficient (ICC) as a measure of clustering where ICC > 0.05 indicated substantial clustering at levels 2 and 3 [21]. In the conditional models 3–5, we also assessed the significance of the change in model deviance as a measure of improved model fit and the increase in R^2^ relative to the unconditional model in step 2 where families were used as random effects. For the fully conditional model in step 5, we calculated the adjusted regression coefficient (B), 95% confidence intervals (CIs), and p values of all fixed effect variables for levels 1 and 2.

## Results

Out of 460 children, complete data were available for 450 (97.8%) children from 246 families in 7 villages with an average of 1.8 children per family and 64.3 children per village. Table 1 shows that the children were 9.9 years old on average, most of them were females (56%), 81.9% did not brush their teeth twice daily and they consumed an average of 3.4 types of sugary foods daily. There were 2.4 siblings on average. Most mothers (87.6%) had less than secondary school education, did not brush their teeth twice daily (85.4%), and consumed an average of 1.4 types of sugary foods daily. The children had a mean of 3.6 teeth with caries experience, 81.9% had caries experience with mean plaque index score = 1.5 and gingival index score = 1.2. There were 135 children who were 12 years and older. Of these, 82 (60.7%) had very low caries experience, 25 (18.5%) had low caries experience, 20 (14.8%) had moderate caries experience, 7 (5.2%) had high caries experience and 1 (0.7%) had very high caries experience. There were 348 children with siblings (77.3%).

**Table 1:** Sociodemographic and oral health practices of children and mothers included in the study

Factors		Children with siblings N = 348	All children N = 450
		N (%)	N (%)
Child level factors			
Age	Mean (SD)	10.0 (2.9)	9.9 (3.0)
Sex	Male: n (%)	151 (43.4)	198 (44.0)
	Female: n (%)	197 (56.6)	252 (56.0)
Child brushing twice daily	Yes: n (%)	63 (18.1)	81 (18.0)
	No: n (%)	285 (81.9)	369 (82.0)
Child sugar consumption daily score	Mean (SD)	3.3 (1.8)	3.4 (1.8)
Family and mother level factors			
Number of siblings in family	Mean (SD)	2.5 (0.8)	2.4 (1.0)
Mother with secondary education or higher	Yes: n (%)	43 (12.4)	56 (12.4)
	No: n (%)	305 (87.7)	394 (87.6)
Mother brushing twice daily	Yes: n (%)	53 (15.2)	66 (14.7)
	No: n (%)	295 (84.8)	384 (85.4)
Mother sugar consumption daily score	Mean (SD)	1.4 (1.0)	1.4 (1.1)
Children oral health status			
Number of teeth with caries experience in child: dmf/ DMF	Mean (SD)	3.5 (3.1)	3.6 (3.1)
Child plaque index	Mean (SD)	1.5 (0.6)	1.5 (0.6)
Child gingival index	Mean (SD)	1.2 (0.4)	1.2 (0.4)

There was no clustering within villages in caries experience (ICC = 0.00), plaque accumulation (ICC = 0.00) or gingival inflammation (ICC = 0.01). All three conditions showed substantial clustering in families (ICC = 0.10, 0.44 and 0.29) in the unconditional model that included families and villages as random effect factors. Therefore, villages were not further included as random effect factors beyond this first unconditional model.

Table 2 shows that, compared to the unconditional model that included families as random effect factors, adding individual (level 1) factors significantly improved the resulting conditional model fit in case of caries experience (p < 0.001) and gingivitis (p < 0.001) but not plaque accumulation (p = 0.90) with 30.1% increase in variation explained in case of caries experience and < 0.00 in case of plaque accumulation and gingival inflammation.

There was no significant improvement in the fit of the resulting conditional model when familial fixed effect factors were added in case of caries experience (p = 0.61), plaque accumulation (p = 0.10), or gingival inflammation (p = 0.20). There was a minimal increase in R2 in the models of caries experience and gingival inflammation (0.12% and < 0.00%) whereas R2 increased by 29.4% in the plaque accumulation model.

Adding level 1 and level 2 fixed effect factors together significantly improved the fit of caries experience conditional model (p < 0.001) and the gingival inflammation model (p < 0.001) with the greatest increase in R2 observed in the caries experience model followed by the plaque accumulation model (increase in R2 = 31.1% and 5.9%) whereas the increase in R2 in the gingival inflammation model was < 0.00%.

**Table 2:** Clustering and model fit of caries experience, plaque accumulation and gingival inflammation

Statistics	dmf/ DMF	PlI	GI
Unconditional model- no fixed effects, families as random effect factor			
-2LL	1544.1	452.2	235.7
Conditional model- Level 1 fixed effect factors			
-2LL	1455.9	451.6	215.3
Improved model fit: X2 (p value)	88.2 (< 0.001)	0.60 (0.90)	20.4 (< 0.001)
%Increase in R2	30.1	< 0	< 0
Conditional model- Level 2 fixed effect factors			
-2LL	1542.3	445.9	231.1
Improved model fit: X2 (p value)	1.80 (0.61)	6.30 (0.10)	4.60 (0.20)
%Increase in R2	0.12	29.4	< 0
Conditional model-Level 1 and level 2 fixed effect factors			
-2LL	1449.9	445.3	210.5
Improved model fit: X2 (p value)	94.2 (< 0.001)	6.90 (0.44)	25.2 (< 0.001)
%Increase in R2	31.1	5.9	< 0

Level 1 (child) fixed effect factors: age, sex, brushing 2x, sugar score

Level 2 (family) fixed effect factors: number of siblings mother’s education, mother’s brushing 2x, mother’s sugar score

Table 3 shows that child’s age was significantly associated with the number of teeth with caries experience: one-year older children had about half a tooth less with caries experience (B= -0.48, p < 0.001) than children of younger age. Children who brushed their teeth twice daily had one more tooth with caries experience than children who did not brush twice daily (B = 1.04, p = 0.01). None of the individual or familial factors was significantly associated with plaque accumulation. Older children had significantly more severe gingival inflammation: one-year older children had a 0.032 higher gingival index score than children who were younger (B = 0.032, p < 0.001).

**Table 3:** association between levels 1 and 2 factors and caries experience, plaque accumulation and gingival inflammation using multilevel linear models

Factors	dmf/ DMF		PlI		GI	
	B (95% CI)	P value	B (95% CI)	P value	B (95% CI)	P value
Child level factors						
Age	-0.48 (-0.59, -0.38)	< 0.001	0.001 (-0.02, 0.02)	0.95	0.032 (0.01, 0.04)	< 0.001
Male vs. female	0.46 (-0.12, 1.05)	0.12	0.02 (-0.08, 0.13)	0.65	-0.01 (-0.09, 0.06)	0.75
Twice daily brushing vs. not	1.04 (0.24, 1.83]	0.01	-0.01 (-0.16, 0.15)	0.95	-0.07 (-0.17, 0.04)	0.22
Daily sugar intake score out of 8	0.02 (-0.14, 0.19)	0.77	-0.008 (-0.04, 0.02)	0.62	-0.001 (-0.02, 0.02]	0.95
Family and mother level factors						
Number of siblings in family	0.17 (-0.23, 0.58)	0.39	0.07 (-0.03, 0.16)	0.15	0.02 (-0.04, 0.07)	0.58
Secondary or higher education vs. less	-0.10 (-0.90, 1.11)	0.84	0.23 (-0.01, 0.46)	0.06	0.14 (-0.003, 0.29)	0.05
Twice daily brushing vs. not	-0.82 (-1.72, 0.09)	0.08	-0.04 (-0.25, 0.17)	0.71	0.07 (-0.06, 0.20)	0.29
Daily sugar intake score out of 8	0.27 (-0.06, 0.60)	0.11	0.03 (-0.04, 0.11)	0.39	0.002 (-0.05, 0.05)	0.95

B: adjusted regression coefficient, CI: confidence interval

## Discussion

The key finding of the study is that in a rural setting in Egypt, there was no substantial clustering of caries experience, plaque accumulation or gingival inflammation in villages. Thus, regardless of the administrative boundaries, these villages showed similar levels of oral conditions. There was a substantial clustering of oral conditions within families and the magnitude of clustering differed by oral condition with greater within-family clustering of plaque accumulation followed by gingival inflammation then caries experience. Individual factors such as child’s age and toothbrushing twice daily explained most of the between-children differences in caries experience. Although child’s age was significantly associated with gingival inflammation, it contributed little to explaining between children’s variation in this condition. None of the individual factors was significantly associated with plaque accumulation nor did they explain between-children variation. Also, none of the familial factors was significantly associated with any of the three oral conditions although familial factors explained some of the random variation in plaque accumulation. Thus, some familial factors not included in the study might have impacted the children and might explain some of their plaque accumulation. The findings, thus, partly support the study hypothesis.

The study is limited by its cross-sectional design which does not prove causality but only suggests associations to be further verified in future longitudinal studies. The study was conducted in rural areas around Alexandria in northwestern Egypt. Although rural areas share similar features in different countries and in the same country, confirmation from other studies is needed from rural areas in other regions in Egypt to allow generalization to a wider scale, especially considering that the majority of the Egyptian population lives in rural areas. In addition, some familial factors were not included in the study and might have affected the oral conditions we studied leaving a portion of the variance unexplained. The study, however, uses a household survey and includes all members of the target population allowing for greater representation. We also included all children in the same household which enabled the assessment of similarities and differences among siblings in contrast to previous studies using the mother-child dyad sampling strategy [22].

Several important findings emerge. First, there was substantial clustering of oral conditions within families but not within villages with possible implications for the design of oral health promotion interventions. Thus, health promotion interventions may address the same level of oral diseases in different villages. Also, with this high level of clustering of plaque accumulation and gingival inflammation in families, it may be efficient to identify families at high risk of these conditions and direct preventive and health promotion activities at them so that the family as a whole receives the intervention allowing interaction, exchange of experiences and mutual support among family members. We provided an estimate of clustering measured by ICC in the study and this would help the design of studies using cluster sampling at family or household levels and allow correct variance estimates to be calculated [23, 24]. It is also important to disentangle the biological, behavioral and contextual factors that may be associated with a higher risk of oral conditions in some families but not others.

Second, one-third of the variation in caries experience was explained by the child’s age and frequent toothbrushing. Older children had less severe caries experience which may be explained by the new eruption of sound permanent teeth replacing decayed primary teeth as children get older. This may suggest inadequate parental attention for their young children’s teeth and oral hygiene and calls for focused dental health education for raising their awareness. On the other hand, older age was associated with significantly more severe gingival inflammation although the clinical significance of greater severity may be minimal: 0.032 points on a scale from zero to 3. Toothbrushing using fluoridated toothpaste is a modifiable oral hygiene practice that was reported to be responsible for caries decline in western countries in the 20th century [25]. Toothbrushing is targeted by behavior modification interventions to improve and maintain oral health [26]. It is important to note, however, that the observed association in the present study indicated that brushing was associated with more, not less, severe caries experience in agreement with previous research conducted in Saudi Arabia [27]. This association may suggest that toothbrushing is a reaction to caries rather than a preventive measure to avoid it [28]. The small percentage of children who brush their teeth twice daily and the high prevalence of caries experienced in the present study further support this interpretation and agree with international surveys showing that the prevalence of twice daily toothbrushing among schoolchildren in Egypt is among the lowest globally [29]. The reactionary nature of toothbrushing is further confirmed by its non-significant and weak association with plaque accumulation in the present study.

Third, plaque accumulation was not significantly associated with any of the individual or familial factors included in the study. Previous research [30] also showed no association between plaque accumulation and age, sex, or dental pain experience of elementary school Iranian children or their parents’ education and occupation. The present study also showed that frequent toothbrushing was not associated with plaque accumulation. This may be because frequency is not the only toothbrushing parameter related to oral health. Other aspects are also associated with plaque accumulation such as toothbrush type, starting age of brushing, and bite abnormalities [31] and these factors were not included in the present study. The present findings also showed that plaque accumulation had greater clustering within families than other oral conditions which may be explained by other familial factors such as mothers’ educational level affecting children’s health behavior [32], parenting practices affecting plaque accumulation [14] and daily toothbrushing [16]. Further research is needed to identify the familial factors which may be linked to plaque accumulation and oral health behaviors among children in this age in rural areas in addition to the factors included in the present study.

## Conclusion

The study showed no clustering of caries experience, plaque accumulation, or gingival inflammation within villages in rural areas near Alexandria, Egypt and substantial clustering within families. There were differences among oral diseases in the clustering degree. Plaque accumulation showed the greatest within-family clustering and caries experience showed the least. Traditional familial factors related to mothers’ education and oral health practices did not explain differences in the three oral conditions. It is important to identify and target at-risk families using health promotion interventions.

## Data Availability

The dataset generated and/or analysed during the current study is not publicly available because it is currently being used to prepare further publications but is available from the corresponding author on reasonable request.
